# Evaluation of a New Porcine Bone Graft on the Repair of Surgically Created Critical Bone Defects in Rat Calvaria: Histomorphometric and Microtomographic Study

**DOI:** 10.3390/jfb13030124

**Published:** 2022-08-23

**Authors:** Ytalo Fernandes, Rafael Mantovani, Danilo Reino, Arthur Novaes, Michel Messora, Luiz Gustavo Sousa, Daniela Palioto, Sergio Scombatti de Souza

**Affiliations:** 1 Department of Oral and Maxillofacial Surgery and Periodontology, School of Dentistry of Ribeirao Preto, University of Sao Paulo, Av do Café, s/n., Ribeirao Preto 14040-904, SP, Brazil; 2 Department of Basic and Oral Biology, School of Dentistry of Ribeirao Preto, University of Sao Paulo, Ribeirao Preto 14040-904, SP, Brazil

**Keywords:** bone regeneration, bone substitute, histology

## Abstract

The aim of this study was to evaluate the use of a new porcine bone graft in rat calvaria bone defects. Critical defects were surgically created in 24 rats that were divided into four experimental groups according to defect filling (*n* = 6): Control Group (CG)—blood clot; Porcine Bone Group (PG)—porcine-derived bone substitute; (BG): Bio-Oss Group (BG)–chemically and heat-treated bovine graft; Bonefill Group (BFG)—chemically treated bovine bone substitute. Euthanasia of the animals occurred 30 days after the surgery, and the area of the original surgical defect and the surrounding tissues were removed for micro-CT and histomorphometric analysis. In the micro-CT evaluation, the PG presented statistically significant differences (*p* < 0.05) in comparison to the CG, BG and BFG, for the parameters percentage of Bone Volume (BV/TV), Surface Bone Density (BS/TV), Number of Trabeculae (Tb.N) and Bone Connectivity (Conn), but not for Total Porosity (Po.tot) and Trabecular Thickness (Tb.Th). The histomorphometric analysis showed that the PG presented similar results to the BG regarding newly formed bone extension and to the BG and BFG regarding newly formed bone area. The porcine-derived graft presented superior microtomographic and histomorphometric results when compared to the two bovine bone substitutes.

## 1. Introduction

Guided bone regeneration has been successfully used in dentistry for different clinical indications. A barrier membrane blocks the migration of undesired cells into the bone defect, and the intact vascular supply collaborates to the osteoconductive process that will repair the alveolar bone [1,2]. Bone growth with the use of grafts occurs through the orientation of osteogenic cells from the existing bone surfaces to the grafted particles, leading to the formation of bone tissue between them, connecting to a mineralized tissue mass. The physical structure of the graft constitutes a framework, allowing the migration of cells and vascular elements that would facilitate bone repair, favoring the osteoconduction [3,4].

An ideal bone grafting biomaterial should present biocompatibility, good stability, be capable of sterilization, easy manipulation, as well as being resorbable and replaceable with new bone tissue [5]. Xenogeneic grafts, biomaterials obtained from other species, usually bovine, have an osteoconductive potential, offering stabilization to the clot in the early stages of the healing, and providing support for new bone repair in the later stages, being chemically and physically similar to human bone [6]. Several studies have shown good results by using xenograft grafts in socket preservation [1,2,7]. For safety reasons, xenogenic materials of bovine origin need to be completely deproteinized through thermal or chemical strategies to eliminate the risk of transmission of bacteria, virus, or prion particles [8]. There are scientific evidences that heating bovine grafts has an effect on its morphological and structural characteristics and can significantly modify its biological performance (osteoconductivity) and crystallinity [9,10].

Although bovine bone grafts are widely used in dentistry, Sogal and Tofe, (2009) assessed the risk of transmission of Bovine Spongiform Encephalopathy (BSE) and concluded that there is a risk of Creutzfeldt–Jacob disease transmission in humans by bovine bone substitutes [11]. In a systematic review by Kim et al. (2011), the risk possibility of the BSE transmission in humans through the use of bone substitutes of bovine origin was highlighted. The authors concluded that bovine graft-derived biomaterials may pose a risk of prion transmission to patients, although the risk cannot be quantified by the currently available data [12]. More recently, Kim et al., in 2016 [13], reported that the ability to track prions within the animal genome is limited, and that the latency period for disease manifestation is long (1 to over 50 years) in infected patients. This fact, according to the researchers, provides a basis for discussing the possible long-term risks of xenografts.

Most porcine tissues and organs are remarkably similar to humans, both structurally and physiologically. This includes several organs, such as the heart, circulatory system, and skin, which is almost indistinguishable histologically [14]. Thus, porcine biomaterials have also been widely used in dentistry, with good preclinical [15] and clinical results [16,17]. Then, the possibility of developing a porcine-originated bone graft, with good biocompatibility and less biological risk when compared to other animal species (mainly to bovine grafts), seems to be a promising alternative for clinical use. Recently, a new particulate bone substitute of porcine origin was developed. The bone tissue for manufacturing the bone substitute is obtained from both medullary and cortical porcine long bones, and later underwent chemical and thermal treatments, resulting in a particulate material with granules of approximately 0.25–1 mm.

Given the advantages of the use of xenogenic grafts in bone repair, and the similarities found between the human and pig tissues, the aim of the present study was to evaluate the results obtained by using a porcine-derived bone substitute in the bone repair of critical surgically created defects in rat calvaria, evaluating microtomographic and histomorphometric parameters.

## 2. Materials and Methods

The present study was approved by the Ethics Committee on Animal Experimentation of the School of Dentistry of Ribeirao Preto-USP (protocol number 2018.1.579.58.0). The procedures were carried out in accordance with the ethical rules governed by the Brazilian College of Animal Experimentation (COBEA).

### 2.1. Sample Size Calculation

The sample size calculation was performed using Statulator, an online statistical calculator [18], to compare the results between two averages of a selected variable. Previous results of our group showed data for selecting the variable area of newly formed bone, with a difference of 1 unit between groups and a standard deviation of 0.5 units. A power of 90%, with a significance level of 5%, a bicaudal test, and groups with the same number of animals were established conditions. The calculated sample size was 6 animals per group. Thus, a total sample size of 24 animals was reached to obtain a significant result.

### 2.2. Experimental Groups

Twenty-four 3-month-old male Sprague Dawley rats, weighing approximately 250–300 g, were selected for the study. The rats had free access to water and a standard diet and were kept in plastic cages. Previously to the surgical procedures, the animals were allowed to acclimatize to the laboratory environment for 7 days. The rats were equally and randomly divided (using a table generated by the website Randomization.com-http://www.randomization.com (accessed on 10 January 2019) into four experimental groups, comprising 6 animals each:

- Control group (CG)—the bone defect was filled only with blood clot, no bone substitutes were used;
- Porcine Bone Group (PG): Experimental group—the bone defect was filled with a new porcine-derived bone substitute (Bonefill Porcinum, Bionnovation, Bauru, São Paulo, Brazil);
- Bio-Oss Group (BG): The bone defect was filled with a chemically and heat-treated inorganic bovine bone substitute (Bio-Oss^®^ Small, Geistlich, Wolhusen, Switzerland);
- Bonefill Group (BFG): The bone defect was filled with a chemically (and not heated) treated inorganic bovine bone substitute (Bionnovation, Bonefill Porous^®^, Bauru, São Paulo, Brazil).

### 2.3. Porcine-Derived Graft Preparation

For graft preparation, the medullary and cortical portions of porcine long bones were used. A physical process with pressurized and heated water jets was initially applied to the bone pieces, complemented with mechanical scraping to remove organic material that was adhered. Afterwards, the bones were cut into smaller sizes to facilitate the separation of the medullary and cortical portions, which were processed following the same sequence of events, but with variations in time (cortical bone, as it is denser, requires longer processing in some steps). Then, the bone fragments, divided into medullary and cortical portions, received a physical processing in a gravitational force equipment and pressurized heated water jet in a closed environment. This equipment accelerates the removal of fat and organic remains through rotation and pressure of the heated water. After that, the final organic remains, such as proteins and fats, were removed through a chemical process, in which the product was washed with strong bases and solutions in different concentrations of alcoholic compounds. Subsequently, the pieces were crushed into smaller granules, between 0.25 and 1 mm of diameter. Following that, the bone received the final chemical treatment, with subsequent abundant washing in water to remove or inactivate all the chemical products previously used. Then, the bone fragments underwent a thermical processing (at 600 °C) to remove organic compounds derived from carbon. Following that, particles without sizes from 0.25 to 1 mm in diameter were removed. Then, the graft underwent quality analysis through standardized tests for the presence of organic remnants. After quality analysis approval, the product was disposed in the final packages with a proportion (by weight) of 70% medullary bone and 30% cortical bone, and finally sterilized by gamma radiation.

### 2.4. Surgical Procedures

**Figure 1:**
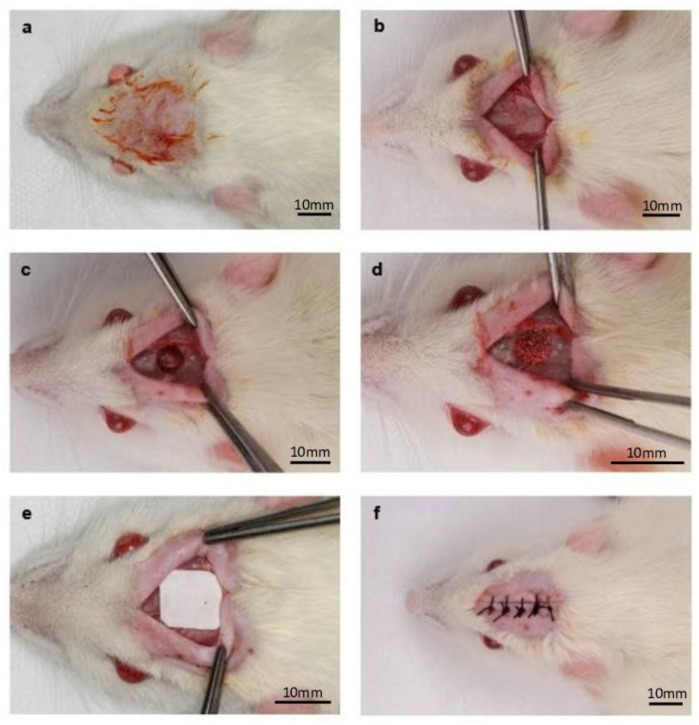
Experimental surgery: (**a**) Skull dorsum after trichotomy and antisepsis; (**b**) Mucoperiosteal flap displaced after incision, exposing the bone tissue; (**c**) Critical Size Defect (CSD) created, with anterior and posterior amalgam marks; (**d**) CSD filled with bone substitute; (**e**) PTFE membrane positioned; (**f**) Flap repositioned and sutured.

The surgical procedures are illustrated in Figure 1. Initially, the animals were anesthetized by intramuscular injection with a 2% Xylazine Hydrochloride solution (2 mg/mL–10 mg/Kg) and Ketamine Hydrochloride a 10% (10 mg/mL–80 mg/KG). After anesthesia, trichotomy and antisepsis of the dorsal region of the skull were performed, using 1% povidone iodine (PVPI). A semilunar incision allowed the reflection of a full thickness flap in a posterior direction and access to the bone tissue. A critical size defect (CSD) of 5 mm in diameter was defined using a 5 mm trephine burr mounted in a low-speed handpiece under continuous and abundant sterile saline solution irrigation (0.9%), preserving the dura mater to maintain the animal’s brain integrity. Circular marks of 2 mm in diameter were made 2 mm anterior and 2 mm posterior to the margins of the surgical defect, using a 5 mm trephine drill (Harte Surgical Instruments, Ribeirão Preto, São Paulo, Brazil), and then filled with amalgam to facilitate the identification of the center of the original bone defect in the histomorphometric analysis. In the bone graft groups, the volume of the inserted biomaterial was standardized in 0.02 mL. In all groups, the bone defect was covered by a teflon membrane (PTFE Surgitime, Bionnovation, Bauru, São Paulo, Brazil) of 10 mm × 10 mm in size.

After that, sutures using absorbable threads (Vicryl Ethicon 5.0, Johnson Prod., São José dos Campos, Brazil) were performed. In the trans-surgical period, intramuscular injections of 2 mg/kg of tramadol hydrochloride (Agner União^®^, Apucarana, PR, Brazil) were administered to produce trans- and postsurgical analgesia, Banamine^®^ 0.2 mL/100 g (Injectable Pet—Schering-Plough, Cotia, SP, Brazil) for anti-inflammatory effect, and 24,000 IU/Kg of Penicillin G-benzatine (0.01 mL) (Pentabiotic* Veterinário Small Size, Fort Dodge Animal Health^®^, Campinas, SP, Brazil) to prevent infections.

Thirty days after the calvaria defects filling, the animals were euthanized with intraperitoneal injections of 10 mg/mL lidocaine (0.7 mg/kg body weight) associated with 2.5% sodium thiopental (Thiopentax^®^, Cristália Produtos Químicos Farmacêuticos Ltd., Itapira, Brazil; 150 mg/kg body weight). The portion within the original surgical defect and the surrounding tissues were removed and fixed in 10% neutral formalin for 24 h, and then transferred to a 70% ethanol solution.

### 2.5. Computed Microtomographic Analysis (Micro-CT)

After fixation, the specimens were scanned with the Skyscan 1172 micro-CT scanner (Bruker, Kontich, Antwerp, Belgium), generating 3D images. For image acquisition, a resolution of 7.9 μm was selected, and the X-ray generator was operated at an accelerated potential of 60 kV with a current of 165 μA. Using the software DataViewer v.1.4.3 (Skyscan N.V.), the generated three-dimensional image was rotated into a standard position for analysis, and then the region of interest (ROI) and the volume of interest (VOI) were delimited. In the calvaria, a 5 mm in diameter ROI, corresponding to the CSD originally created, was determined. The VOI was calculated as a cylindrical figure determined by ROI and a standard height of 0.5 mm corresponding to the thickness of the calvaria. The cylindrical figure was positioned in the region of the defect so that: (a) 0.25 mm of its height involved the portion located between the dura mater and the center of the defect and (b) 0.25 mm of its height involved the portion located between the center of the defect and the external surface of the calvaria [19].

**Figure 2:**
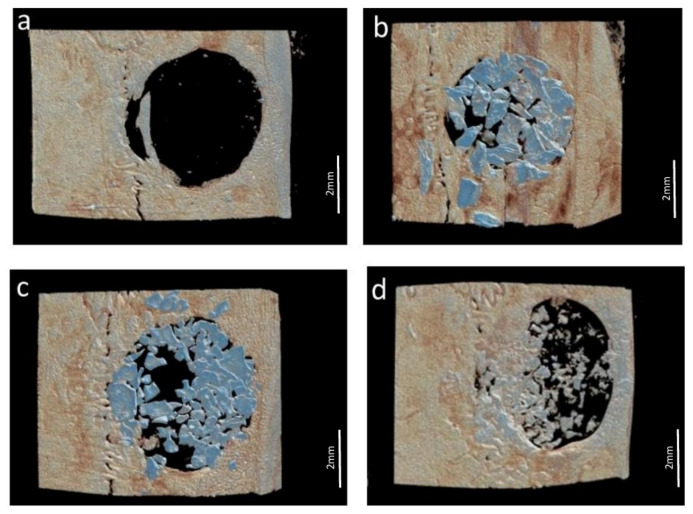
Micro-CT analysis—three-dimensional reconstructions of the defects. (**a**) Control Group; (**b**) Porcine Group; (**c**) Bio-Oss Group; (**d**) Bonefill Group.

The following parameters were analyzed in each VOI by a calibrated examiner (Y.F.F.) using the CT-Analyser^®^ v.1.13.5.1+ software (Bruker, Kontich, Belgium): Bone surface density (BS/TV), defined as the ratio between the bone surface area and the total volume of the VOI, expressed in 1/mm; bone volume percentage (BV/TV), defined as the ratio between bone volume and the total volume of the VOI, expressed in percentage (%); trabecular thickness (Tb.Th), defined as the average thickness of the trabeculae, expressed in mm; number of trabeculae (Tb.N), defined as the average number of trabeculae per mm, expressed in 1/mm; total Porosity (Po.tot), defined as the percentage of pores per analyzed VOI; bone connectivity (Conn), defined as a measure of the degree to which a structure is multiply connected and the maximum number of connections that can be broken before the structure is separated into two parts. Illustrative 3D reconstructions of each group are shown in Figure 2.

### 2.6. Histomorphometric Analysis

After the micro-CT scanning, the specimens were rinsed with water and decalcified in 4% ethylenediaminetetraacetic acid solution. After decalcification, each specimen was divided longitudinally into two blocks exactly along the center line of the original surgical defect using the amalgam marks as reference, and after that, processed and embedded in paraffin in a process standardized with the Histo Embedder (Leica Reichert & Jung Products—Heildelberg, Germany). Then, a series of 5µm thick sections were cut in a longitudinal direction starting at the center of the original surgical defect. Then, the sections were deparaffinized, hydrated and stained with hematoxylin–eosin (H.E.) and Masson’s Trichrome (T.M.) for light microscopy analysis. Two nonserial histological sections (one of each block) of each animal were selected. Each histological section was captured by a brightfield fluorescence microscope with trinocular head and 1.6 objective (model DMLB, Leica Microsystems, Wetzlar, Germany) connected to a camera (DFC300FX, Leica Mycrosystems, Wetzlar, Germany).

The histomorphometric analysis was performed by a single blinded calibrated examiner (Y.F.F.) using an image acquisition and analysis software (LAS EZ version 4.1.0, Leica Mycrosystems^®^). The intraclass correlation coefficient (ICC) was used to determine the examiner’s reproducibility. ICC values greater than 90% were considered to ensure examiner calibration. The data obtained was normalized and analyzed using the GraphPad Prism 5.0 statistical software. The evaluated parameters included: total area (TA) of the originally created defect, in mm^2^; newly formed bone area (NFB), expressed in percentage (%) of the TA; total extension (ESD) of the original surgical defect in mm; and newly formed bone extension (NBE), expressed in mm and in percentage. The area of the remaining particles (ARP), expressed in percentage (%) of the TA was also measured for the bone substitute groups.

### 2.7. Statistical Analysis

The animal was considered the statistical unit. The Lilliefors normality test indicated the normality of the data. Analysis of variance (ANOVA) test followed by Tukey’s subtest were used for between groups comparisons. The Bioestat software (BioEstat, Version 5.3, Instituto de Desenvolvimento Sustentável Mamirauá, Tefé, Brazil) was used, and the significance level was established at 5% for all evaluations (*p* < 0.05)

## 3. Results

### 3.1. Porcine and Bovine Derived Graft Characterization

**Figure 3:**
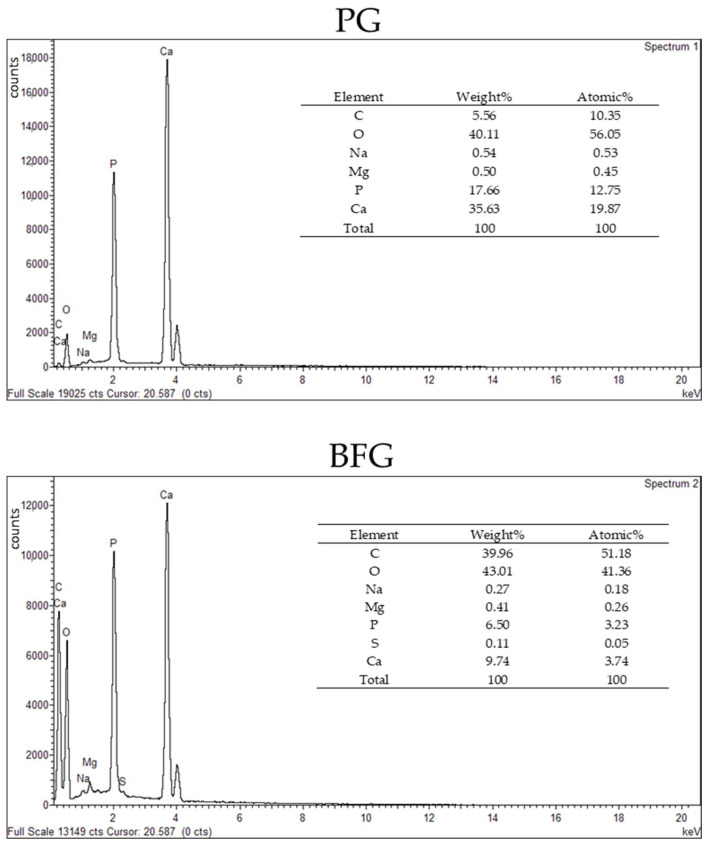
EDS from the Porcine Group (PG) and Bonefill Group (BFG) graft samples.

The grafts used in the PG and BFG groups were structurally and chemically characterized. To identify and characterize the elemental composition present on the grafts, energy dispersive X-ray spectroscopy (EDS) measurements were performed using an INCAx-Sight System; (Oxford Instruments, Abingdon, UK) at a 15 kV voltage, spot 5.0, and a working distance of 10 mm (Figure 3). The PG presented higher percentages (both in weight and atomic composition) of Ca (35.36% in weight) and P (17.66% in weight) when compared to the BFG (Ca = 9.74%; *p* = 6.50% in weight). The PG also presented lower percentages of C (5.56% in weight and 10.35% in atomic) in comparison to the BFG (39.96% in weight and 51.18% in atomic).

**Figure 4:**
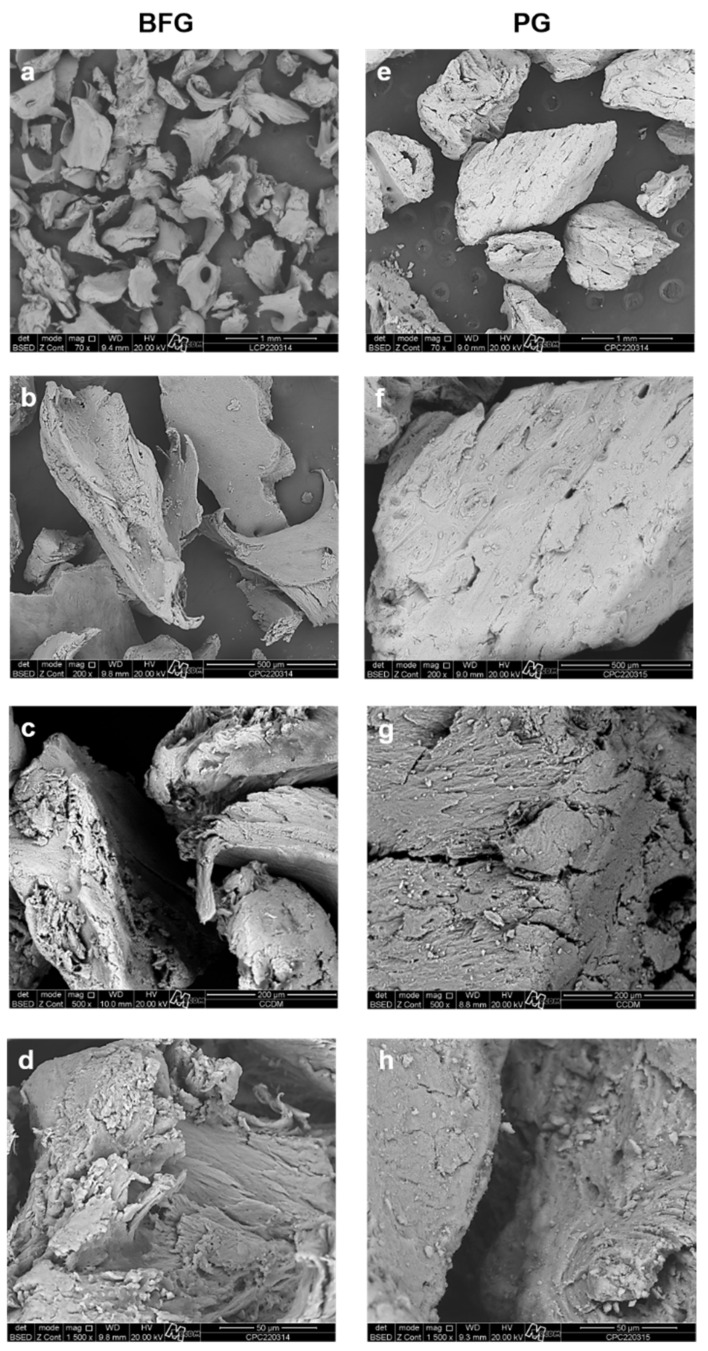
High-resolution scanning electron micrographs of the BFG (**a**–**d**) and PG (**e**–**h**) showing ultrastructural surface characteristics of the biomaterials, with a magnification of 70× (**a**,**e**), 200× (**b**,**f**), 500× (**c**,**g**), and 1500× (**d**,**h**).

A Quanta 400 FEG D8630 Scanning Electron Microscope (SEM) (FEI Company, Thermo Fisher, Waltham, MA, USA, EUA) was used to analyze the ultrastructural morphology of these surfaces, and the representative scanning electron micrographs are shown in Figure 4. The BFG presented smaller particles and, in higher magnification, a rougher surface, when compared to PG. The images also suggested that the PG particles had more porosity than the BFG.

### 3.2. Micro-CT Results

**Figure 5:**
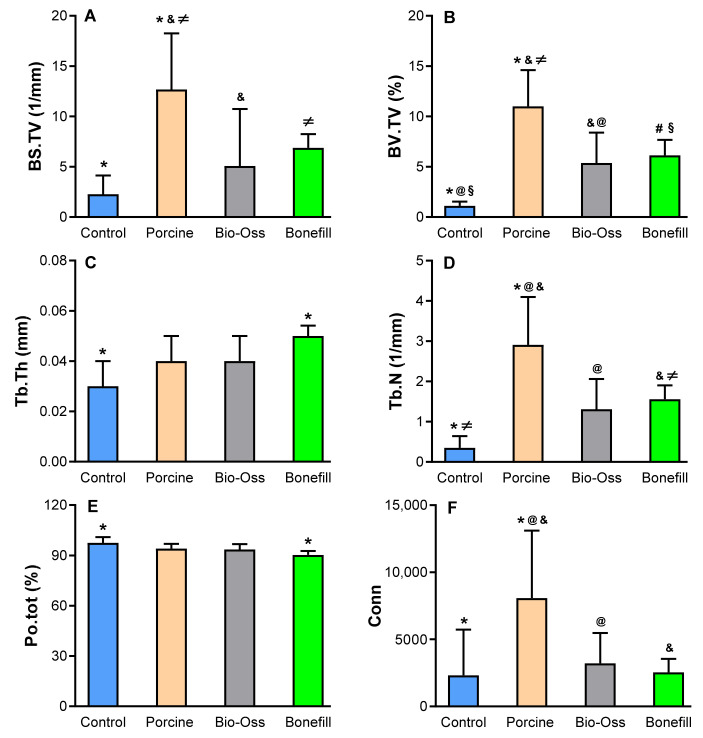
Micro-CT results of the critical size defects in rat calvaria: mean and standard deviations (SD) of bone surface density (**A**) (BS/TV, 1/mm); bone volume percentage (**B**) (BV/TV, %); trabecular thickness (**C**) (Tb.Th, mm); number of trabeculae (**D**) (Tb.N, 1/mm); total porosity (**E**) (Po.tot, %); bone connectivity (**F**) (Conn) for each experimental group. Equal symbols indicate statistically significant differences between groups (*p* < 0.05): * = Control versus Porcine; @ = Control versus Bio Oss; § = Control versus Bonefill; & = Porcine versus Bio Oss; # Porcine versus Bonefill.

The micro-CT results are summarized in Figure 5. Regarding BS/TV, there were statistically significant differences between the PG (12.69 ± 5.57) versus the CG (2.27 ± 1.85), BG (5.08 ± 2.66), and BFG (6.87 ± 3.38) (Figure 5A). In the analysis of BV/TV (Figure 5B), statistically significant differences were observed for the PG (10.99 ± 3.61) versus the CG (1.11 ± 0.41), BG (5.38 ± 3.02), and BFG (6.14 ± 3.75). There were also statistically significant differences for the BG and BFG versus the CG.

Regarding Tb.Th (Fibure 5C), the values were: CG (0.03 ± 0.01), PG (0.04 ± 0.01), BG (0.04 ± 0.01), and BFG (0.05 ± 0.01). There was a statistically significant difference only between the BFG versus the CG. In the analysis of Tb.N (Figure 5D), there were statistically significant differences between the PG (2.91 ± 1.19) versus the CG (0.35 ± 0.29), BG (1.31 ± 0.75) and BFG (1.56 ± 0.84), with the highest average number of trabeculae for the PG. There was also a statistically significant difference between the BFG versus the CG.

For Po.tot (Figure 5E), the values for each group were: CG (97.48 ± 3.46), PG (94.03 ± 2.88), BG (93.56 ± 3.21), and BFG (90.32 ± 5.70). Only the GC versus the GBF showed statistically significant difference. In the bone connectivity analysis (Figure 5F), statistically significant differences were observed between the PG (8087.29 ± 5020.51) versus the CG (2327.29 ± 3397.65), BG (3204.00 ± 2265.24), and BFG (2534.00 ± 2499.15).

### 3.3. Histomorphometric Analysis

**Figure 6:**
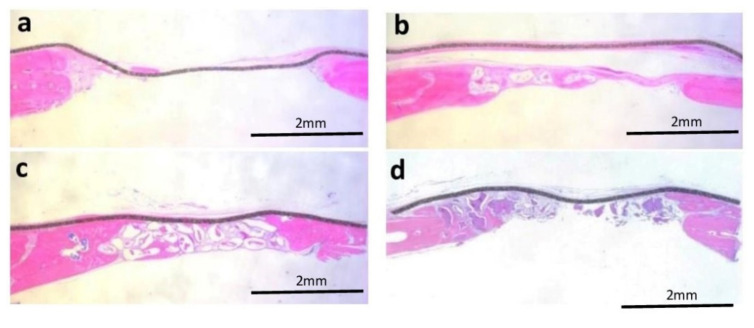
Panoramic histological images of all experimental groups at 1.6× magnification, stained with hematoxylin and eosin (H.E): (**a**) Control Group; (**b**) Porcine Group; (**c**) Bio-Oss group; (**d**) Bonefill Group.

**Figure 7:**
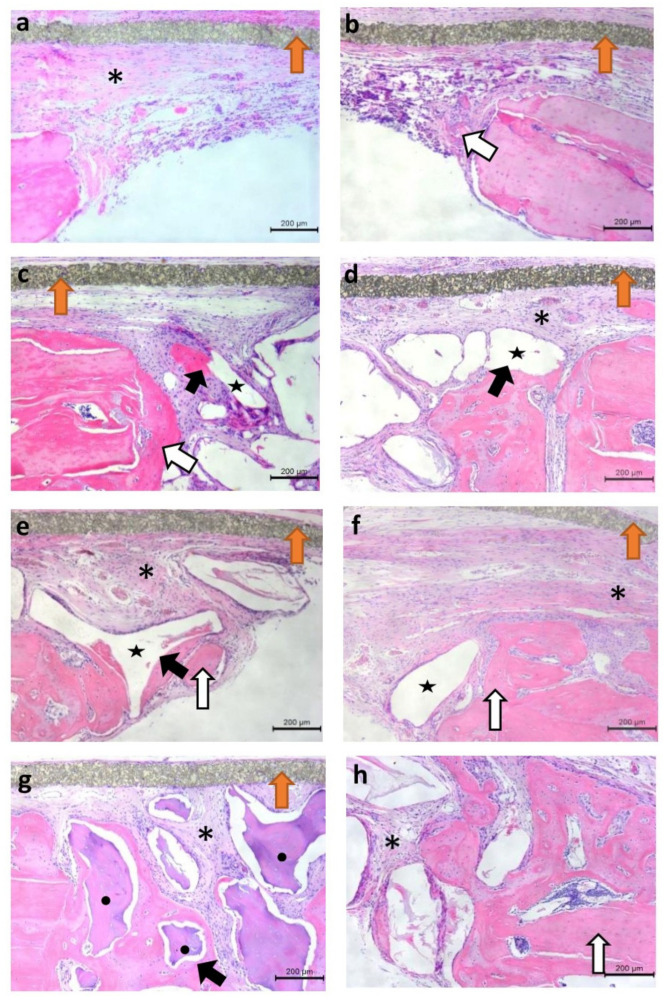
Histological images of the experimental groups at 10× magnification. Slides stained with hematoxylin and eosin (H.E.). (**a**,**b**) Edges of the defect in the Control Group; (**c**,**d**) The Porcine Group defect; (**e**,**f**) Edges of the defect in the Bio-Oss group; (**g**,**h**) Edges of the defect in Bonefill group. **Black arrows**: Direct contact between the biomaterial particles and the newly formed bone. **White arrows**: Bone tissue. **Orange arrows**: PTFE membrane. **Black Stars**: Remaining particles of the biomaterial. **Asterisks**: Area of nonmineralized fibrous tissue.

Panoramic photomicrographs of all analyzed groups are shown in Figure 6, and more detailed images, in higher magnification, can be seen in Figure 7. In the analysis of the total area (TA, in mm^2^), statistically significant differences were found between the the PG (5.14 ± 0.34) versus the CG (4.59 ± 0.41), BG (4.53 ±0.42), and BFG (4.47 ± 0.32). For the area of newly formed bone (NFB, in mm^2^), there was a statistically significant difference between the CG (2.98 ±2.76) versus the PG (7.86 ± 3.99), BG (6.30 ± 3.62), and BFG (8.94 ± 7.40), with higher values for the groups in which bone substitutes were used. No statistically significant differences were found between the PG, BG, and BFG.

The analysis of the linear extension of the surgical defect (in mm) showed that there were no statistically significant differences between the experimental groups (CG = 5.02 ± 0.01, PG = 5.03 ± 0.01, BG = 5.12 ± 0.18, and BFG = 5.02 ± 0.01). For the newly formed bone extension (NBE, in mm), statistically significant differences were observed between the PG (0.77 ± 0.30) versus the CG (0.40 ± 0.10) and BFG (0.43 ± 0.07), but not versus the BG (0.67 ± 0.35).

Regarding the parameter of the area of remaining particles (ARP, in mm^2^), which was calculated as a percentage of TA in the bone substitute groups, no statistically significant differences were found between the PG (12.41 ± 11.38), BG (8.84 ± 6.36), and BFG (9.76 ± 5.06).

## 4. Discussion

The present study evaluated the bone repair after the use of an experimental porcine-derived bone graft compared to two different bovine bone substitutes. A critical size defect (CSD) in rat calvaria was used as the experimental model, since it has been characterized in the literature as one of the most used preclinical models to assess the regenerative potential of biomaterials [20,21], in addition to showing physiological similarity to human bone remodeling [22,23,24].

Bone regeneration is an important goal in implant dentistry. Alveolar bone reconstruction is often required to allow installation of implants in a correct tridimensional position, and the use of a bone substitute biomaterial associated to a membrane to guide the bone regeneration is frequently required for a successful treatment. The membrane blocks the epithelial and gingival connective tissue cells and also helps to stabilize the graft at the surgical site. In the present study, the membrane was used in all groups, as its absence could lead to an increased rate of biomaterials reabsorption [25], and as in agreement with previous reports [26,27], the grafted groups presented the highest volumes of bone formation.

Although there are many relevant studies evaluating the use of bovine bone graft for guided bone regeneration, there are scarce publications comparing the use of porcine versus bovine bone grafts. Park et al., in 2016 [28], evaluated, in an in vivo model in rabbit calvaria, the osteogenic activity of four different types of bone substitutes: porcine-derived bone graft (PD), porcine-derived bone graft with the addition of strontium ions (ES/Sr), phosphate particles from synthetic micro/macroporous biphasic calcium (BCP), and particulate demineralized allogeneic lyophilized bone graft (DA), with evaluations after 3 and 6 weeks. In the histological and histomorphometric analyses, the PD presented NFB in the margins of the defect, but with the presence of fibrous tissue around the remaining granules in the central area. The ES/Sr presented the highest bone neoformation in the central portion of the defect among all analyzed groups. In the 6-week evaluation, the BV/TV was 12.5% for the PD, and this result is in agreement with the values obtained in the present study (PG: BV/TV 10.99%). The values for the ES/Sr, BCP, and DA groups were 16.7%, 17.2%, and 27.3%, respectively. Although the DA group had the highest mean for BV/TV, it also showed large variations between specimens (12.9–61.8%), compromising its reliability. In the present study, the PG presented the highest BV/TV values, when compared to the other three groups.

Kim et al., in 2018 [29], compared in rat calvaria defects the bone formation in hybrid graft of porcine-derived bone substitute versus Bio-Oss. Micro-CT analysis at 4 and 8 weeks showed superior bone formation in the grafted groups compared to the control group (no biomaterial, only blood clot) without statistical difference of the BS/TV and BV/TV between the porcine-derived group and the Bio-Oss group, while in the present study, there was a statistical difference between the PG and BG for BS/TV and BV/TV (*p* =< 0.05), favoring the PG. In addition, in the 4-week histomorphometric analysis, the porcine-derived group presented more angiogenesis and less remaining particle area (ARP) when compared to the Bio-Oss group. In the present study, there were no significant differences in relation to the ARP between the groups in which bone substitutes were used, suggesting that all three groups, PG, BG, and BFG, presented similar resorption attributes. The results of Kim et al. also showed that both grafts showed statistically significant differences for increased bone volume compared to the control group. These results are in agreement with the present study: there were significant differences for the NFB between the CG and the three grated groups.

In a 2019 study, Bae et al. [30] evaluated the regenerative capacity of a bone graft of porcine origin versus a bone graft of bovine origin (Bio-Oss) in rat calvaria. In the micro-CT analysis for BV/TV after 4 weeks, both grafts showed positive results for newly formed bone, with a slight tendency for the porcine graft, but without statistically significant differences—the values obtained were 17.52 ± 3 0.88% for the porcine graft and 11.6 ± 3.88% for the bovine graft. Although this study did not find significant differences between the groups, these results agree with those found in the present study, in which the micro-CT analysis showed higher values with a significant difference for the BV/TV in the PG compared to the BG (10.99 ± 3.61% and 5.38 ± 3.02%, respectively). Furthermore, the NBF values showed in the study by BAE et al., 2019, after 4 weeks for the swine group (9.08 ± 5.47) and the bovine group (5.83 ± 2.56), showed higher numerical values for the porcine group, although without statistically significant difference; these results were similar to those found in the present study (7.86 ± 3.99 for the PG, and 6.30 ± 3.62 for the BG), thus showing similar capacities of bone neoformation for both groups.

In the present study, all groups with the bone graft showed better BV/TV results when compared to the control group. Among the groups with bone graft, better results were observed for the GS (10.99 ± 3.61) versus the GB (5.38 ± 3.02) and GBF (6.14 ± 3.75%). The results obtained for the GB are in agreement with the findings of Park et al., 2009 [31], with the BV/TV values of 6.4% for the Bio-Oss. Leventis et al., in 2018 [32], evaluated bone defects in rabbit calvaria treated with a synthetic biomaterial for Bio-Oss at 8 weeks postoperatively, and found similar results between groups for the BV/TV, with significantly higher (33.10 ± 8.94 for the synthetic graft and 33.10 ± 8.94) values than that found in the present study. These differences may be explained by the use of different animal species (rabbit versus rat) and sacrifice periods (8 weeks versus 4 weeks).

In the histomorphometric analysis of the present study, regarding NFB (7.86 ± 3.99 mm^2^) and NBE (0.77 ± 0.30 mm), the porcine bone group presented statistically superior results when compared to the CG (NFB = 2.98 ± 2.76 mm^2^; NBE = 0.40 ± 0.10 mm). The results of new bone formation obtained for the groups with the use of bone substitutes (PG = 7.86 ± 3.99 mm^2^, BG = 6.30 ± 3.62 mm^2^, BFG = 8.94 ± 7.40 mm^2^) were numerically similar to that presented by the Bio-Oss in the study by Park et al., 2009 [31], (NFB 6.4 ± 4.3 mm^2^) after 6 weeks in critical defects in rats calvaria, showing comparable bone formation 2 weeks prior to this period (4 weeks for the present study).

Among the limitations of the present study are the short period of evaluation, which did not allow analyzing the behavior of the biomaterials in a longer term. Another limitation was the low bone formation presented in all the studied groups: this phenomenon may be associated with the animal model used. It is known that critical defects in rat calvaria may present some characteristics related to the metabolism of the region, including poor vascularization and fragment instability [33,34]. However, it is important to emphasize that the order of magnitude of the results was similar to that already reported in the literature for the same type of defect and bone substitute [30,31]. Additionally, the scarce number of articles addressing the use of porcine-derived bone substitutes limited the discussion of the results obtained in comparison to previous reports: further studies are needed, including longer evaluation periods.

Osteogenesis, osteoconduction, rapid resorption, and biodegradation are desired features of bone substitutes. The results of the present study suggest that all the evaluated biomaterials showed biocompatibility and osteoconduction, as well as predictability for bone formation in the created defect, when associated with the PTFE membrane. The porcine-derived bone substitute showed superiority in relation to the control group filled with blood clot and presented similar properties to those presented by bovine-derived grafts, primarily regarding neoformed bone area and extension, as well as superior results regarding bone density, bone volume, the number of trabeculae, and the trabecular connectivity, thus showing it to be a viable biomaterial option for use in guided bone regeneration.

## 5. Conclusions

The porcine-derived bone substitute presented superior microtomographic and histomorphometric results when compared to the two bovine bone substitutes. Furthermore, the two bone grafts of bovine origin did not differ from each other regarding all evaluated parameters. These results suggest that the porcine-derived bone substitute tested in rat critical calvaria defects offers a favorable cellular response, and a bone regeneration capacity similar to that produced by the bovine substitutes currently available.

## Data Availability

The data presented in this study are available on request from the corresponding author. The data are not publicly available due to ethical reasons.
